# Integration of Transcriptomics and WGCNA to Characterize *Trichoderma harzianum*-Induced Systemic Resistance in *Astragalus mongholicus* for Defense against *Fusarium solani*

**DOI:** 10.3390/genes15091180

**Published:** 2024-09-08

**Authors:** Jingping Niu, Xiang Yan, Yuguo Bai, Wandi Li, Genglong Lu, Yuanyuan Wang, Hongjun Liu, Zhiyong Shi, Jianping Liang

**Affiliations:** 1 College of Life Sciences, Shanxi Agricultural University, Taigu, Jinzhong 030801, China; niujingping@sxau.edu.cn (J.N.); yanx0715@163.com (X.Y.); 15837071231@163.com (Y.B.); 13835937031@163.com (W.L.); lglong1313@126.com (G.L.); w13327549040@163.com (Y.W.); m15971755763@163.com (H.L.) zhiyong-shi@163.com (Z.S.); 2 Modern Research Center for Traditional Chinese Medicine, Shanxi University, Taiyuan 030006, China

**Keywords:** *Astragalus mongholicus*, *Trichoderma harzianum*, *Fusarium solani*, RNA-Seq, WGCNA, RT-qPCR

## Abstract

Beneficial fungi of the genus *Trichoderma* are among the most widespread biocontrol agents that induce a plant’s defense response against pathogens. *Fusarium solani* is one of the main pathogens that can negatively affect *Astragalus mongholicus* production and quality. To investigate the impact of *Trichoderma harzianum* on *Astragalus mongholicus* defense responses to *Fusarium solani*, *A. mongholicus* roots under *T. harzianum* + *F. solani* (T + F) treatment and *F. solani* (F) treatment were sampled and subjected to transcriptomic analysis. A differential expression analysis revealed that 6361 differentially expressed genes (DEGs) responded to *T. harzianum* induction. The Kyoto Encyclopedia of Genes and Genomes (KEGG) pathway enrichment analysis of the 6361 DEGs revealed that the genes significantly clustered into resistance-related pathways, such as the plant–pathogen interaction pathway, phenylpropanoid biosynthesis pathway, flavonoid biosynthesis pathway, isoflavonoid biosynthesis pathway, mitogen-activated protein kinase (MAPK) signaling pathway, and plant hormone signal transduction pathway. Pathway analysis revealed that the *PR1*, formononetin biosynthesis, biochanin A biosynthesis, and *CHIB*, ROS production, and *HSP90* may be upregulated by *T. harzianum* and play important roles in disease resistance. Our study further revealed that the H_2_O_2_ content was significantly increased by *T. harzianum* induction. Formononetin and biochanin A had the potential to suppress *F. solani*. Weighted gene coexpression network analysis (WGCNA) revealed one module, including 58 DEGs associated with *T. harzianum* induction. One core hub gene, *RPS25*, was found to be upregulated by *T. harzianum*, SA (salicylic acid) and ETH (ethephon). Overall, our data indicate that *T. harzianum* can induce induced systemic resistance (ISR) and systemic acquired resistance (SAR) in *A. mongholicus*. The results of this study lay a foundation for a further understanding of the molecular mechanism by which *T. harzianum* induces resistance in *A. mongholicus*.

## 1. Introduction

*Astragalus membranaceus* (Fisch.) *Bge.* var. *mongholicus* (Bge.) Hsiao (Leguminosae) is a medicinal plant that is distributed across China, Japan, Korea, and Southeast Asia [1]. Astragalus root is a well-known traditional Chinese medicine that is listed in the Chinese Pharmacopoeia [2]. And it has rich medicinal properties, such as antioxidant, tonic, hepatoprotectant, antidiabetic, and anticancer properties [3]. However, owing to the expanded cultivation area and continuous cropping, *A. membranaceus* is prone to infection by various phytopathogens in the rhizospheric soil, which severely affects its yield and quality [4]. Astragalus root rot caused by *Fusarium* spp. is the most common disease of *A. membranaceus* [5]. Severe Astragalus root rot can reach an incidence of more than 80% and even lead to a complete lack of harvest [6]. The use of the resistant cultivar HQN03-03 [5], crop rotation [5,7], and a combination of fungicide and insecticide [8] have been reported to control Astragalus root rot to some extent. However, these protection measures are time-consuming, involve excessive labor costs, can harm human health and the environment [9], and lead to the development of fungicide-resistant strains of pathogens [10]. For these reasons, biocontrol agents (BCAs) offering a sustainable ecological solution for disease management [11] are needed. *Trichoderma* is considered to be the largest genus of BCAs, comprising 25 species, and is the most commonly used genus for controlling phytopathogenic fungi [12]. Importantly, *Trichoderma* spp. have diverse biocontrol traits, including parasitism, antibiosis, and the production of secondary metabolites (SM), and can cause the induction of the plant’s defense system [13]. *Trichoderma reesei* has been shown to inhibit the growth of *F. solani* and effectively prevent the occurrence of Astragalus root rot [14]. The induction of plant defense mechanisms against pathogens via the application of biological control agents (BCAs) is a novel approach to achieving plant protection [15,16]. However, this mechanism has not been reported in *Trichoderma* spp.-induced resistance to *A. membranaceus.*

The two main mechanisms, including induced systemic resistance (ISR) and systemic acquired resistance (SAR), are identified in activating the defense mechanisms in plants [17]. Resistance associated with the colonization of plant roots by certain plant-growth-promoting rhizobacteria (PGPR) is called ISR [18]. ISR is effective against a broad spectrum of plant pathogens and relies mainly on the jasmonic acid (JA) and ethylene (ET) signaling pathways [17,19]. SAR is typically initiated by local infection, offers long-term systemic resistance against subsequent pathogen attacks, is associated with the activation of PR genes, and demands the participation of the signal molecule salicylic acid (SA) [20]. *Trichoderma* spp. has been reported to induce a mixed ISR or SAR against *Rhizoctonia solani* [21]. The expression of genes related to ISR-dependent ethylene and jasmonate (ET/JA) and SAR-dependent salicylic acid (SA)-mediated signaling pathways in tomatoes was upregulated by the *T. harzianum* strain M10 [22]. Moreover, genes involved in the oxidative stress response, the phenylpropanoid pathway [23], terpenoid metabolites [24], etc., were also shown to be associated with *Trichoderma* spp.

In the resistance study of Astragalus root rot, only one article mentioned that JA content and ISR-related enzyme activities can be induced by a simplified bacteria synthetic community [25]. It is insufficient to mine resistance genes of *A. membranaceus* at the genome level. In recent years, transcriptome analysis and weighted gene coexpression network analysis (WGCNA) have been employed to understand the transcriptomic changes in host tissue during the early phases of *Trichoderma*–host interaction [26,27]. And WGCNA can be used to ascertain the correlation between module eigengenes and hub genes in relation to sample traits [28]. Nevertheless, to our knowledge, transcriptome and WGCNA analysis of *T. harzianum* inducing resistance of *A. mongholicus* to *F. solani* have not been reported. To explore the mechanism by which *T. harzianum* induces *A. mongholicus* resistance to *F. solani* in this study, differential gene expression between the *T. harzianum* + *F. solani* treatment and *F. solani* treatment groups was analyzed at the transcriptional level. Subsequently, key KEGG pathways were analyzed on the basis of the differentially expressed genes (DEGs). Finally, weighted gene coexpression network analysis (WGCNA) was performed to identify the hub genes, and the expression of the hub genes was analyzed via hormone induction. These findings could enrich the gene resources of *A. mongholicus* resistant to *F. solani* and lay a foundation for future research on *Trichoderma* spp. inducing *A. membranaceus* resistance.

## 2. Materials and Methods

### 2.1. Plant Material and T. harzianum and F. solani Inoculation

Seeds of susceptible material *A. mongholicus* were obtained from Hunyuan County, Shanxi Province, China. *T. harzianum* T9131 and *F. solani* HYFS-1, which were isolated from *A. mongholicus* plants with root rot, were grown on potato dextrose agar (PDA) plates and incubated in darkness for 7 days at 27 °C.

Seeds were planted in 14 cm-diameter plastic pots containing nutrient soil and vermiculite (volume ratio of 1:1). Nutrient soil and vermiculite were purchased from GREEN HOPE (Linyi, ShanDong, China) and were sterilized for one hour at 121 °C using an oven (JH881-3, Junhuan Machinery, Suzhou, Jiangsu, China). A total of 10–15 seedlings were planted in each pot, and the pots were subsequently placed in a climate-controlled cabinet at 25 °C with a 16 h light/8 h dark photoperiod. Half of the *A. mongholicus* plants grown for one month were inoculated with fresh spore suspensions of *T. harzianum* (T) (conc. 10^8^ spores mL^−1^) by application on the roots via watering (about 10 mL for each plant), and the plants inoculated with water alone were used as controls. Then, all the plants were put back in the climate-controlled cabinet. All the *A. mongholicus* plants were subsequently inoculated with *F. solani* (F) via the hypocotyl inoculation technique [29] after T treatment for 48 h and subsequently placed in a high-humidity mist chamber for 24 h. *A*. *mongholicus* roots were collected at 0, 24, and 48 h after F infection, and three replicates were examined.

### 2.2. RNA Sequencing and Functional Annotation

A total of 18 RNA samples, representing T + F and F treatments in triple biological repeats, were extracted with a TRIzol reagent kit (Invitrogen, Carlsbad, CA, USA) following the manufacturer’s instructions. The purity and concentration of the RNA were determined via a Nanodrop2000 (Thermo Fisher Scientific, Waltham, MA, USA). The integrity of the RNA was detected via agarose gel electrophoresis, and RNA integrity (RIN) values were determined with an Agilent 5300 Bioanalyzer (Agilent Technologies, Santa Clara, CA, USA). The RNA-seq transcriptome libraries of 18 samples were prepared at Shanghai Majorbio Biopharm Biotechnology Co., Ltd. (Shanghai, China) following ligation by Illumina (San Diego, CA, USA) and sequenced via the Illumina NovaSeq 6000 platform.

A large number of raw reads were produced via Illumina NovaSeq 6000 sequencing. Subsequently, the raw paired-end reads were trimmed and subjected to quality control by employing FASTP (v0.19.5) (https://github.com/OpenGene/fastp (accessed on 5 December 2023)) with default parameters. Trinity software (v2.8.5) (https://github.com/trinityrnaseq/trinityrnaseq/wiki (accessed on 5 December 2023)) [30] was used for de novo assembly of the clean reads of the 18 samples. The assembly results were evaluated by employing TransRate (v1.0.3) (http://hibberdlab.com/transrate/ (accessed on 7 December 2023)) [31]. The integrity of the assembly was assessed by means of BUSCO (v3.0.2) (http://busco.ezlab.org (accessed on 7 December 2023)). The annotation of the unigenes obtained through Trinity assembly was performed using the following six databases: the Non-Redundant Protein Sequence Database (NR) (ftp://ftp.ncbi.nlm.nih.gov/blast/db/ (accessed on 15 January 2024) [32], Gene Ontology (GO) (http://www.geneontology.org (accessed on 15 January 2024) [33] and Kyoto Encyclopedia of Genes and Genome (KEGG) (http://www.genome.jp/kegg/ (accessed on 15 January 2024)) [34], Evolutionary Genealogy of Genes: Non-supervised Orthologous Groups (EggNOG) (http://www.ncbi.nlm.nih.gov/COG/ (accessed on 15 January 2024) [35], Swiss-Prot (http://web.expasy.org/docs/swiss-prot_guideline.html (accessed on 15 January 2024)) [36], and Pfam (http://pfam.xfam.org/ (accessed on 15 January 2024) [37]. 

### 2.3. Differentially Expressed Gene Analysis

The gene abundance of each transcript was quantified with RSEM software [38]. The quantitative index was the number of fragments per kilobase per million reads (FPKM). Three pairwise comparisons were conducted based on RNA-seq data, including T + F_0 h vs. F_0 h, T + F_24 h vs. F_24 h, and T + F_48 h vs. F_48 h. The DEGs among different comparisons were identified via DESeq2 software [39]. Genes with a fold change (FC) ≥ 2 and an FDR-adjusted *p* value < 0.05 were considered significant DEGs. Venn diagrams were constructed by using the online tool of the Majorbio cloud platform (https://cloud.majorbio.com/page/tools/ (accessed on 1 February 2024) [40]. KEGG pathway enrichment analysis of the DEGs was implemented via KOBAS [41]. A *p* value < 0.05 was considered to indicate a significantly enriched functional pathway.

### 2.4. Weighted Gene Co-Expression Network Analysis

WGCNA was performed via the WGCNA R software package [42]. To improve the accuracy of the WGCNA, genes with a low expression (FPKM < 1) were removed. The β value was determined by the adjacency matrix using WGCNA. The adjacency matrix was weighted based on the power of correlation data between different genes, and the β value was determined by applying the average connectivity criterion and scale-free topology [43]. The module was identified according to the β value, and the threshold for merging similar modules was 0.25 (mergeCutHeight = 0.25). The module correlating with *T. harzianum* was calculated via the Spearman method; *p* < 0.01 was used as the criterion for screening related modules. The coexpression network was visualized via Cytoscape [44]. 

### 2.5. RT-qPCR Analysis

The template cDNA of the RNA-Seq samples was synthesized via MonScript^TM^ RTIII Super Mix with dsDNase (Monad Biotech., Suzhou, Jiangsu, China) following a standard protocol. The gene primers used for RT–qPCR were designed with Primer Premier 5.0. The housekeeping gene *Am18S* [45] was employed as a reference control in the RT–qPCR analysis of the RNA-Seq samples. The volume and program settings for RT–qPCR were adjusted according to the specifications of MomAmp^TM^ Chemohs qPCR (Monad Biotech., Suzhou, Jiangsu, China). The RT–qPCR was subsequently conducted via a Bio-Rad CFX96 system. Three technical replicates were conducted for each sample. The relative expression levels of eight randomly selected genes from the top five pathways, including plant-pathogen interaction, flavonoid biosynthesis, isoflavonoid biosynthesis, MAPK signaling pathway, and phenylpropanoid biosynthesis pathways, were calculated via the relative 2^−ΔΔCT^ method for validating RNA-Seq results. The relative expression levels of six connected hub genes were also calculated via the relative 2^−ΔΔCT^ method. Information on the primers used is shown in Table S1.

### 2.6. H_2_O_2_ Content Assays

The roots of the T + F and F groups of *A. mongholicus* were collected at 0, 24, and 48 h. And the roots were washed in deionized distilled water (ddH_2_O) and stored in a deep freezer (−80 °C) for H_2_O_2_ content assays. Three replicates were performed. The H_2_O_2_ content was determined via a kit from Sangon Biotech Co., Ltd. (D799773-0050; Shanghai, China) following the manufacturer’s instructions. The H_2_O_2_ content was measured by determining the absorbance at a wavelength of 415 nm via an ultraviolet–visible spectrophotometer (UV-1100, Molecular Devices, Tianjin, China) [28].

### 2.7. Antagonistic Effects of Formononetin and Biochanin A on F. solani In Vitro

Formononetin (purity > 99%, CAS No.: 485-72-3) and biochanin A (purity > 99%, CAS No.: 491-80-5) were purchased from Med ChemExpress (MCE, Shanghai, China). The growth of *F. solani* was inhibited on a PDA medium supplemented with different concentrations of formononetin (0, 5, 10, or 15 mg/mL) or biochanin A (0, 1, 2, 3, or 5 mg/mL), with 0 mg/mL as a control. An *F. solani* colony with a diameter of 6 mm was placed on the middle of the plate and incubated at 27 °C for 6 days, and three replicate experiments were performed per concentration. The percent inhibition of *F. solani* was calculated by using the formula ((control colony diameter − treated colony diameter)/(control colony diameter − 6) × 100) [4]. 

### 2.8. Analysis of RPS25 Expression under Hormone Treatments

SA (purity ≥ 99.5%, CAS No.: 69-72-7), ETH (purity ≥ 95%, CAS No.: 16672-87-0), and MeJA (purity ≥ 95%, CAS No.: 39924-52-2) were purchased from Solarbio Science & Technology Co., Ltd. (Beijing, China). *A*. *mongholicus* plants grown for one month were sprayed with SA (400 µmol/L), ETH (400 µmol/L) and MeJA (200 µmol/L) at a rate of 5 mL per plant, respectively. Roots were sampled at 0, 24, and 48 h after spraying, and three replicates were performed. Total RNA was extracted using the EZ-10 DNAaway RNA Mini-Preps Kit (Sangon Biotech, Shanghai, China) with reference to the manufacturer’s protocol. The cDNA of RNA samples was synthesized via MonScript^TM^ RTIII Super Mix with dsDNase (Monad Biotech., Suzhou, Jiangsu, China) by following a standard protocol. The cDNA was used for the RT–qPCR analysis of gene *RPS25*. 

### 2.9. Statistical Analysis

IBM SPSS Statistics 23 was used for the statistical analysis. A one-way analysis of variance (ANOVA) was carried out to assess the effects of the treatments, and Duncan’s multiple range tests were employed to determine the significant difference at *p* values ≤ 0.05. In all cases, the residuals were subjected to a normality test using the Holm–Sidak test. Figure processing was performed via GraphPad Prism 8. 

## 3. Results

### 3.1. Reduction in the Plant Wilt Rate 

**Figure 1:**
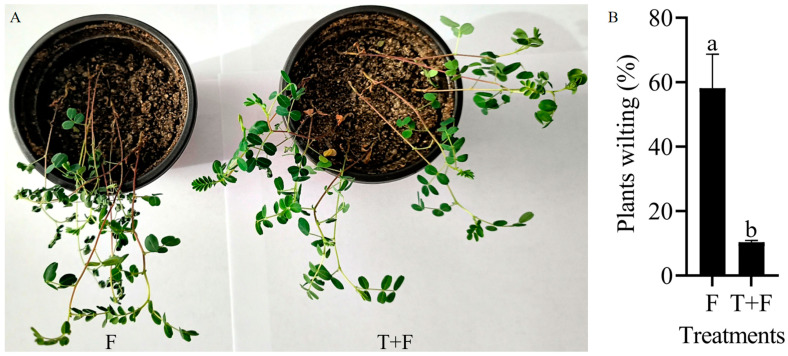
Response of *A. mongholicus* to *F. solani* under *T. harzianum* treatment. (**A**) Plant phenotype. (**B**) Plant wilting rate. F, plant infected with *F. solani*; T + F, plant infected with *F. solani* after *T. harzianum* treatment for 48 h. The error bars represent ±SD values, and different letters indicate significant differences between the two columns (*p* < 0.05).

When *A. mongholicus* plants were infected with *F. solani*, the leaves began to wilt at 48 h [29]. Treating *A. mongholicus* with *T. harzianum* could significantly reduce the onset of symptoms. Approximately 58.18% of the plants exhibited leaf wilting at 48 h after inoculation with *F. solani*, and an average of 10.37% of the plants treated with *T. harzianum* exhibited leaf wilting at 48 h after inoculation with *F. solani* (Figure 1). We speculated that *T. harzianum* could promote the resistance of *A. mongholicus* to *F. solani*.

### 3.2. Transcriptomic Analysis and Identification of Differentially Expressed Genes 

To assess the impact of *T. harzianum* on the *A. mongholicus* defense response against *F. solani*, transcriptome sequencing was performed with *A. mongholicus* from the T + F and F treatment groups at three time points (0, 24, and 48 h). Approximately 48,101,033 clean reads were generated on average (Table S2). The Q20 and Q30 percentages of each sample were greater than 98.23% and 94.65%, respectively (Table S2). The average GC content of all the samples was 42.51% (Table S2). An average of 19,849,736 clean reads were mapped to the transcriptome assemblies. A total of 99,541 unique genes were described, and their expression levels were quantified according to the expected FPKM values.

**Figure 2:**
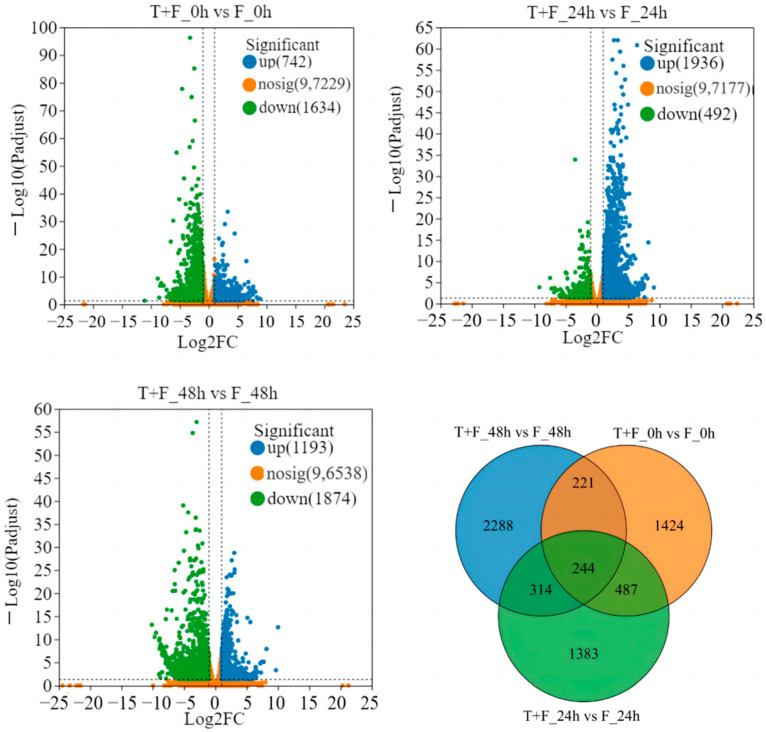
Volcano diagram and Venn diagram of differentially expressed genes (DEGs) between pairs of samples (i.e., T + F_0 h vs. F_0 h, T + F_24 h vs. F_24 h, and T + F_48 h vs. F_48 h). The x-axis shows the fold change in gene expression between samples, and the y-axis represents the statistical test result for the difference in gene expression. The blue dots represent the significantly upregulated DEGs, whereas the green dots represent the significantly downregulated DEGs. The orange dots represent the DEGs that were not significantly differentially expressed.

To identify DEGs that responded to *T. harzianum* treatment, transcriptome comparisons (T + F_0 h vs. F_0 h, T + F_24 h vs. F_24 h, T + F_48 h vs. F_48 h) were conducted. A total of 2376 DEGs (742 DEGs upregulation and 1634 DEGs downregulation), 2428 DEGs (1936 DEGs upregulation and 492 DEGs downregulation), 3067 DEGs (1193 DEGs upregulation and 1874 DEGs downregulation) with a fold change ≥ 2 and *p* adjusted < 0.05 were identified in T + F_0 h vs. F_0 h, T + F_24 h vs. F_24 h, and T + F_48 h vs. F_48 h, respectively (Figure 2). The Venn diagram showed that a total of 6361 DEGs were found in three transcriptome comparisons (Figure 2, Table S3).

### 3.3. Functional Annotation and KEGG Pathway Enrichment Analysis of DEGs

To evaluate the potential functional categories of the DEGs, 6361 DEGs were subjected to GO and KEGG annotation analyses. GO annotation analysis revealed that 4224 of the 6361 DEGs could be classified into 47 functional categories, including 20 biological processes, 12 cellular components, and 15 molecular function terms (Table S4). The top six annotation terms were “binding”, “catalytic activity”, “cellular process”, “metabolic process”, “cell part”, and “membrane part” (Figure S1A). KEGG annotation analysis of 1156 of the 6361 DEGs revealed that the main enriched pathways were “carbohydrate metabolism” and “translation” (Figure S1B).

**Figure 3:**
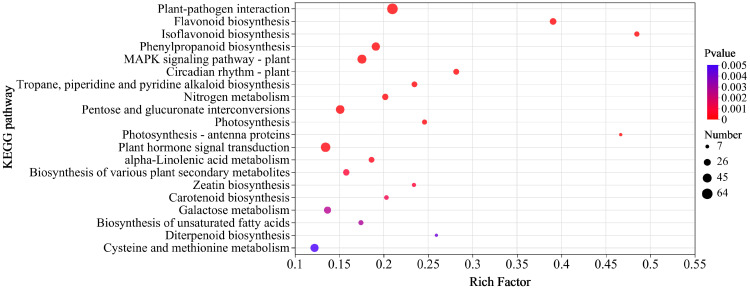
KEGG enrichment analysis of the 6361 DEGs. The x-axis indicates the ratio of the number of DEGs in the pathway to all DEGs. The y-axis indicates the KEGG pathway.

To further explore the gene-regulation pathways, a KEGG pathway enrichment analysis using a *p* value < 0.05 was performed on the 6361 DEGs. The results revealed that the top five enriched pathways were “plant–pathogen interaction” (map04626) [46], “flavonoid biosynthesis” (map00941) [47], “isoflavonoid biosynthesis” (map00943) [48], “phenylpropanoid biosynthesis” (map00940) [49], and “MAPK (mitogen-activated protein kinase) signalling pathway” (map04016) [50] (Figure 3) and were associated with disease resistance. In addition, the plant hormone signal transduction pathway (map04075) [51] related to resistance was also enriched (Figure 3).

### 3.4. Validation of RNA-Seq Data by RT–qPCR

To validate the RNA-Seq results, eight genes from the above top five pathways were randomly selected for RT–qPCR, and the overall expression trends were consistent with the RNA-Seq data for all the genes. The results revealed that the RNA-Seq results were reliable (Figure S2).

### 3.5. Plant–Pathogen Interaction Pathway Analysis under T. harzianum Treatment

**Figure 4:**
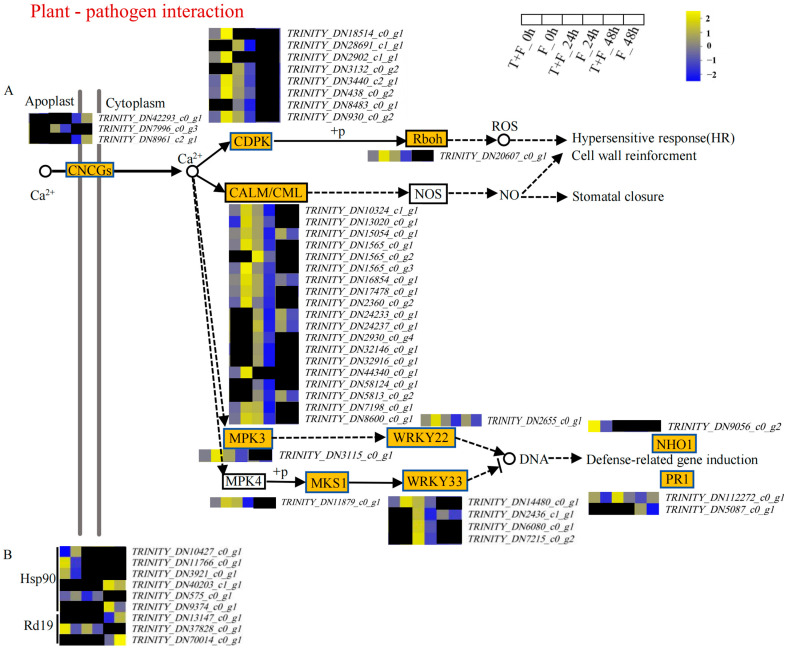
Plant–pathogen interaction pathway information for the DEGs. (**A**) Ca^2+^ signaling pathway-related DEGs; (**B**) other key DEGs. The log_2_FPKM values are indicated by colors. Yellow, upregulated; blue, downregulated; black, no significant difference in expression in the comparison group.

In this study, 41 Ca^2+^ signaling-related DEGs were enriched in the plant–pathogen interaction pathway (Figure 4A, Table S5). These genes were grouped into ten subfamilies, namely cyclic nucleotide-gated ion channels (CNGCs), calcium-dependent protein kinases (CDPKs), calmodulin (CaM) and CaM-like proteins (CMLs), respiratory burst oxidase homolog protein (Rboh), mitogen-activated protein kinase (MPK3), WRKY transcription factor 22 (WRKY22), MAP kinase substrate 1 (MKS1), WRKY transcription factor 33 (WRKY33), nonhost resistance 1 (NHO1), and PR protein 1 (PR1). In T + F_0 h vs. F_0 h, only the expression of *NHO1*- and *PR1*-related genes was significantly upregulated. In T + F_24 h vs. F_24 h, DEGs related to *CNGCs*, *CDPKs*, *Rboh*, *CaM*/*CMLs*, *MPK3*, *WRKY22*, *MKS1*, *WRKY33*, and *PR1* were enriched, and their expression was significantly upregulated. In T + F_48 h vs. F_48 h, the expression of DEGs encoding *CaM/CMLs*, *WRKY22*, *WRKY33*, and *PR1* (*TRINITY_DN5087_c0_g1*) was significantly upregulated.

In addition, DEGs encoding Hsp90 and Rd19 were also enriched (Figure 4B, Table S5). In T + F_0 h vs. F_0 h, the genes *TRINITY_DN11766_c0_g1* (*Hsp90*), *TRINITY_DN3921_c0_g1* (*Hsp90*), and *TRINITY_DN37828_c0_g1* (*Rd19*) were significantly upregulated. *TRINITY_DN37828_c0_g1* (*Rd19*) was significantly upregulated in T + F_24 h vs. F_24 h. *TRINITY_DN9374_c0_g1* (*Hsp90*) was significantly upregulated in T + F_48 h vs. F_48 h. 

### 3.6. Assay of H_2_O_2_ Levels in the T + F and F Treatments

**Figure 5:**
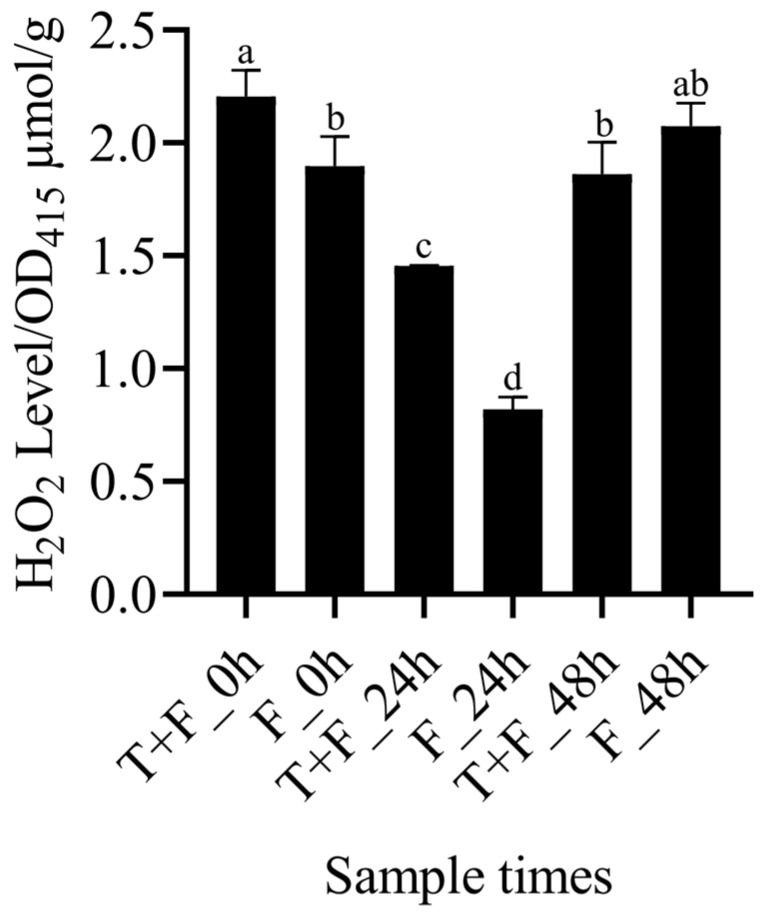
Determination of H_2_O_2_ content in the T + F and F treatments. Error bars represent ±SD values, and different letters indicate significant differences between the two columns (*p* < 0.05).

To confirm the role of H_2_O_2_ in the resistance of *A. mongholicus* to *F. solani*, H_2_O_2_ levels were measured in the T + F and F treatments. The results revealed that H_2_O_2_ levels were significantly greater in the T + F treatment than in the F treatment at 0 h, indicating that H_2_O_2_ accumulation was induced by *T. harzianum* treatment for 48 h. Compared with that at 0 h, the H_2_O_2_ content was significantly lower in the T + F and F treatments at 24 h. Compared with that at 24 h, the H_2_O_2_ content was significantly higher in the T + F and F treatments at 48 h. The H_2_O_2_ content in the T + F treatment was lower than the F treatment at 48 h, and no significant difference was found between the two treatments (Figure 5). 

### 3.7. Formononetin and Biochanin A Biosynthesis Pathway Analysis under T. harzianum Treatment 

**Figure 6:**
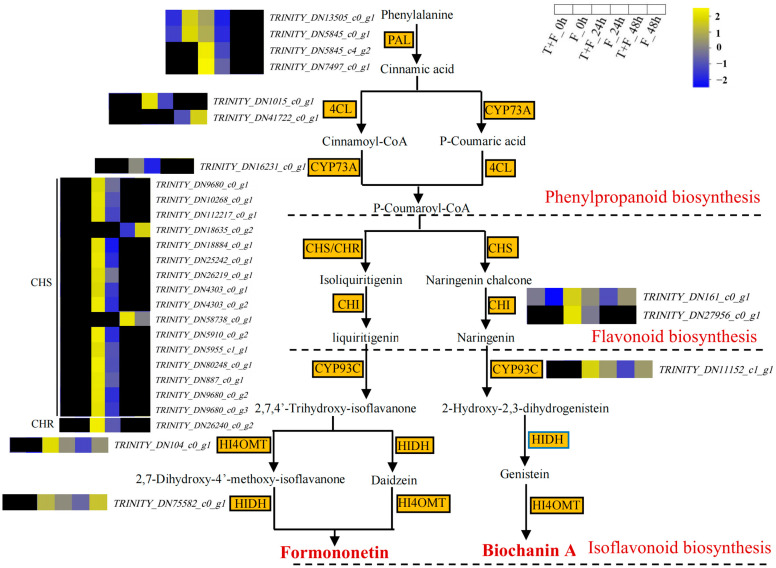
Formononetin and biochanin A biosynthesis pathway information for the DEGs. The log_2_FPKM values are indicated by colors. Yellow, upregulated; blue, downregulated; black, no significant difference in expression in the comparison group at the same time point.

In this study, 29 DEGs related to the formononetin and biochanin A biosynthesis pathways were identified (Figure 6, Table S5). These genes were grouped into eight subfamilies, namely phenylalanine ammonia lyase (PAL), 4-coumarate-CoA ligase (4CL), trans-cinnamate 4-monooxygenase (YCP73A), chalcone synthase (CHS) and chalcone reductase (CHR), chalcone isomerase (CHI), 2-hydroxyisoflavanone synthase (YCP93C), isoflavone 4′-O-methyltransferase (HI4OMT), and 2-hydroxyisoflavanone dehydratase (HIDH). In T + F_0 h vs. F_0 h, only the expression of *TRINITY_DN161_c0_g1* (*CHI*) was significantly upregulated. In T + F_24 h vs. F_24 h, the expression of DEGs related to formononetin and biochanin A synthesis, such as *PAL*, *4CL*, *YCP73A*, *CHS* and *CHR*, *CHI*, *YCP93C*, and *HI4OMT*, was significantly upregulated, and the expression of DEGs related to another key synthesis gene, *HIDH*, was also upregulated. In T + F_48 h vs. F_48 h, the expression of only *TRINITY_DN58738_c0_g1* (*CHS*) was significantly upregulated. 

**Figure 7:**
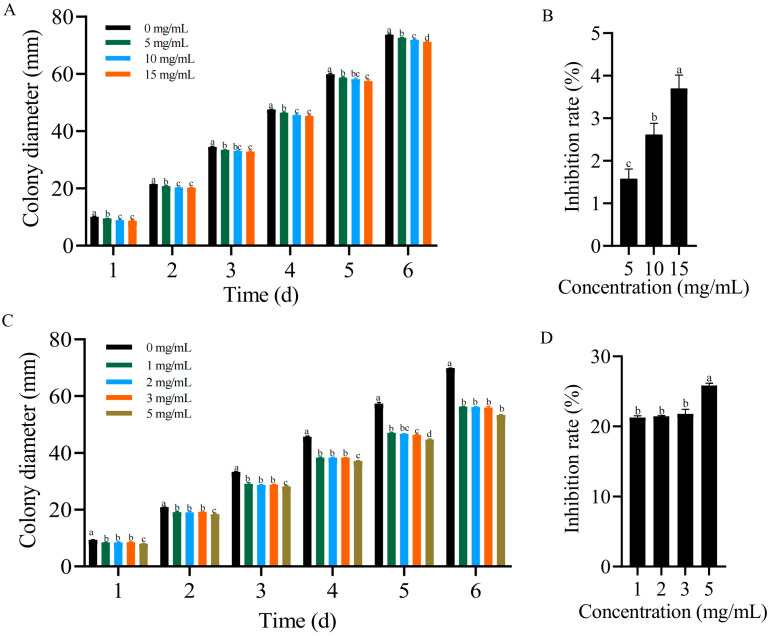
Antifungal activity of formononetin and biochanin A against *F. solani* in vitro. (**A**) Colony diameter after formononetin treatment. (**B**) Mycelial growth inhibition rate after formononetin treatment. (**C**) Colony diameter after biochanin A treatment. (**D**) Mycelial growth inhibition rate after biochanin A treatment. The error bars represent ±SD values, and different letters indicate significant differences between the two columns (*p* < 0.05).

In order to preliminarily determine the formononetin and biochanin A inhibitory potential against *F. solani*, plate inhibition experiments were performed in vitro. Formononetin treatments significantly inhibited the growth of *F. solani* in a dose-dependent manner (Figure 7A). The rate of mycelial growth inhibition was 3.70% when the concentration of formononetin was 15 mg/mL (Figure 7B). Biochanin A treatment also significantly inhibited the growth of *F. solani*. There was no significant difference in the inhibition ability in the 1–3 mg/mL concentration range, and the average inhibition rate was 21.49% (Figure 7C). When the concentration was increased to 5%, the inhibition rate increased to 25.8% (Figure 7D). 

### 3.8. MAPK Signalling Pathway and Plant Hormone Signal Transduction Pathway Analysis under T. harzianum Treatment

**Figure 8:**
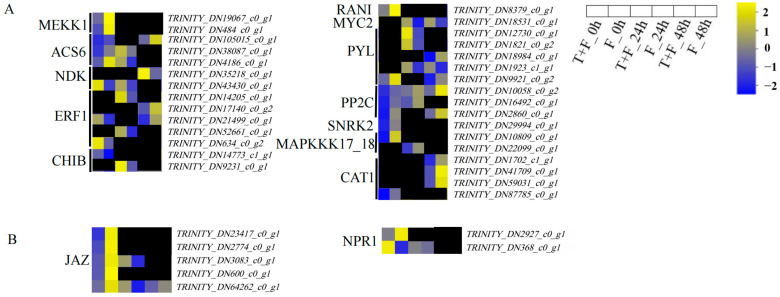
MAPK signaling pathway and plant hormone signal transduction pathway information of the DEGs. (**A**) DEGs in the MAPK signaling pathway; (**B**) DEGs in the plant hormone signal transduction pathway. The log_2_FPKM values are colored. Yellow, upregulated; blue, downregulated; black, no significant difference in expression in the comparison group at the same time point.

In this study, 32 DEGs were enriched in twelve subfamilies in the MAPK signaling pathway (Figure 8A, Table S5). The twelve subfamilies were as follows: mitogen-activated protein kinase kinase kinase 1-like (MEKK1), 1-aminocyclopropane-1-carboxylate synthase (ACS6), nucleoside-diphosphate kinase (NDK), ethylene-responsive transcription factor 1 (ERF1), basic chitinase (CHIB), copper transporter responsive to antagonist 1 (RAN1), myelocytomatosis transcription factor (MYC2), abscisic acid receptor PYL family (PYL), protein phosphatase 2C (PP2C), serine/threonine-protein kinase SRK2 (SNRK2), mitogen-activated protein kinase kinase kinase 17/18 (MAPKKK17_18), and catalase (CAT1). Among these, ERF1, MYC2, PYL, PP2C, and SNRK2 are also involved in plant hormone signal transduction. In T + F_0 h vs. F_0 h, *TRINITY_DN43430_c0_g1* (*NDK*), *TRINITY_DN21499_c0_g1* (*ERF1*), *TRINITY_DN634_c0_g2* (*ERF1*), and *TRINITY_DN14773_c0_g1* (*CHIB*) were significantly upregulated. In T + F_24 h vs. F_24 h, DEGs in the *ACS6*, *NDK*, *ERF1*, *CHIB*, *MYC2*, and *PYL* subfamilies were significantly upregulated. In T + F_48 h vs. F_48 h, *TRINITY_DN35218_c0_g1* (*NDK*), *TRINITY_DN18534_c0_g1* (*MYC2*), and *TRINITY_DN1923_c1_g1 (PYL)* were significantly upregulated. 

In addition, the JASMONATE-ZIM domain (JAZ) and nonexpressor of PR genes1 (NPR1) were enriched in plant hormone signal transduction (Figure 8B, Table S4). In T + F_0 h vs. F_0 h, *TRINITY_DN368_c0_g1* (*NPR1*) was significantly upregulated. In T + F_24 h vs. F_24 h, the DEGs of the *JAZ* and *NPR1* subfamilies were significantly upregulated. 

### 3.9. Gene Coexpression Network Analysis

**Figure 9:**
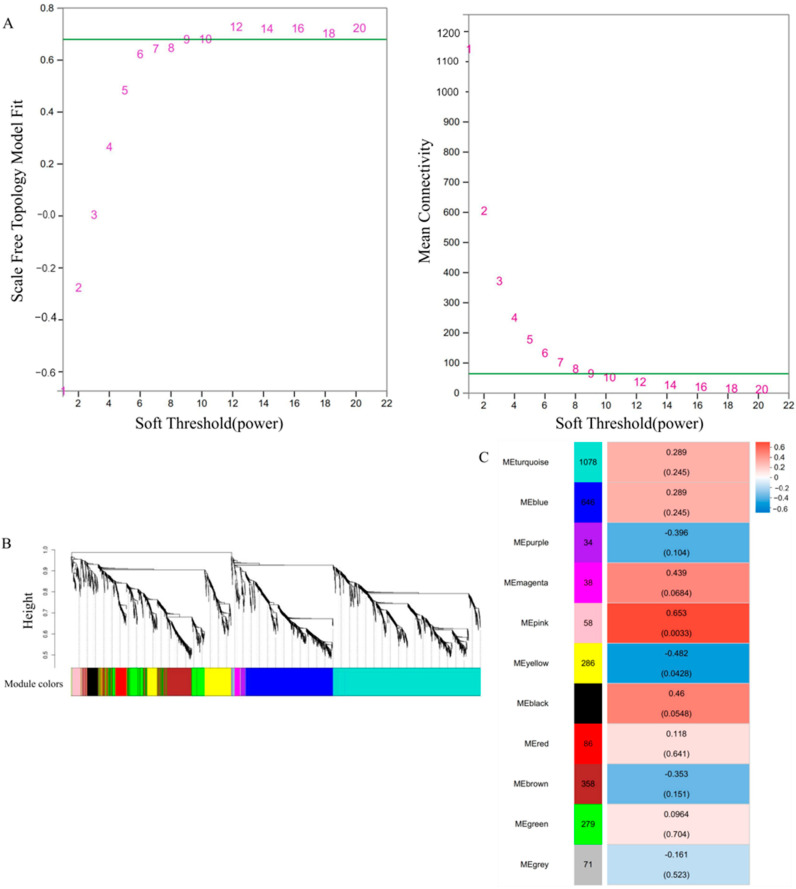
Soft threshold power (β value) determination by WGCNA and module detection. (**A**) Soft threshold determination. The number corresponding to the green line is the most appropriate β value. (**B**) Gene-cluster dendrogram and module colors. (**C**) Correlations between modules and the resistance trait. The correlation coefficients are shown above, and the *p* values are shown below.

Coexpressed gene modules were determined via WGCNA on the basis of 4251 DEGs from the GO and KEGG annotations. When the β value was nine (Figure 9A), 10 modules with module sizes ranging from 34 to 1078 genes and 71 genes outside 10 modules were classified into the grey module (Figure 9B). Module eigengenes were evaluated for their correlation with *A. mongholicus* plants in the T + F treatment. The results revealed that the pink module (R = 0.653, *p* = 0.0033) eigengenes were significantly (*p* < 0.01) positively correlated with each other (Figure 9C). There were 58 genes in the pink module.

**Figure 10:**
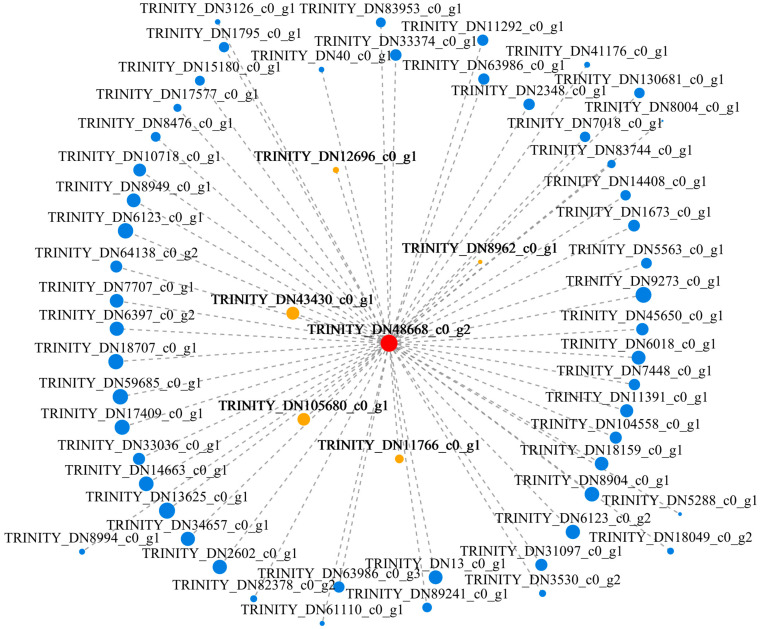
Coexpression networks of the 58 connected hub genes in the pink module related to the T + F treatment. The red circle represents the core hub gene *TRINITY_DN48668_c0_g2*, and the yellow circles represent the coexpressed hub genes *TRINITY_DN43430_c0_g1*, *TRINITY_DN11766_c0_g1*, *TRINITY_DN8962_c0_g1*, *TRINITY_DN12696_c0_g1*, and *TRINITY_DN105680_c0_g1*.

To screen key genes, the hub genes were identified via a coexpression network of the pink module. One hub gene, *TRINITY_DN48668_c0_g2* (*RPS25*), encoding the 40S ribosomal protein S25-2-like, had the highest module membership value. We then constructed a visual depiction of the coexpression network of this hub gene and found that the coexpressed genes included genes encoding nucleoside diphosphate kinase (NDK) (*TRINITY_DN43430_c0_g1*) in the MAPK signaling pathway, heat-shock protein 90-like (Hsp90) (*TRINITY_DN11766_c0_g1*) in plant-pathogen interaction pathway, cyclin D3 (CYCD3) (*TRINITY_DN8962_c0_g1*) related to *Fusarium oxysporum* resistance [52], leucine-rich repeat receptor-like protein kinase (LRR-RLK) (*TRINITY_DN12696_c0_g1*) related to resistance [53], and cytochrome p450 (CYPs) (*TRINITY_DN105680_c0_g1*) related to *Botrytis cinereal* and *Phytophthora parasitica* resistance [54] (Figure 10, Table S6). 

### 3.10. Expression Analysis of Hub Genes and Coexpressed Genes under T. harzianum Treatment

**Figure 11:**
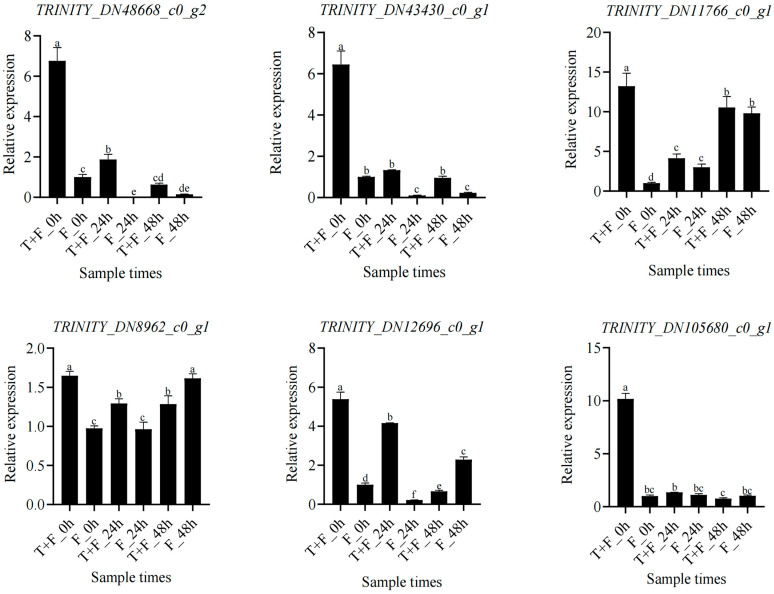
Relative expression analysis of hub genes and coexpressed genes in the T + F and F treatments. The error bars represent ±SD values, and different letters indicate significant differences between the two columns (*p* < 0.05).

To confirm that the above hub genes and the coexpressed genes were indeed induced by *T. harzianum*, an RT–qPCR was performed. The expression of the hub gene *TRINITY_DN48668_c0_g2* (*RPS25*) and the coexpressed genes *TRINITY_DN43430_c0_g1 (NDK*) and *TRINITY_DN11766_c0_g1* (*Hsp90*) was higher in the T + F treatment group than in the F treatment group overall. In addition, the expression of the coexpressed genes *TRINITY_DN8962_c0_g1* (*CYCD3*), *TRINITY_DN12696_c0_g1* (*LRR-RLK*), and *TRINITY_DN105680_c0_g1* (*CYPs*) was greater in the T + F treatment than in the F treatment at 0 h and 24 h (Figure 11). 

### 3.11. Expression Analysis of RPS25 Induced by the Exogenous Hormones SA, ETH, and MeJA

**Figure 12:**
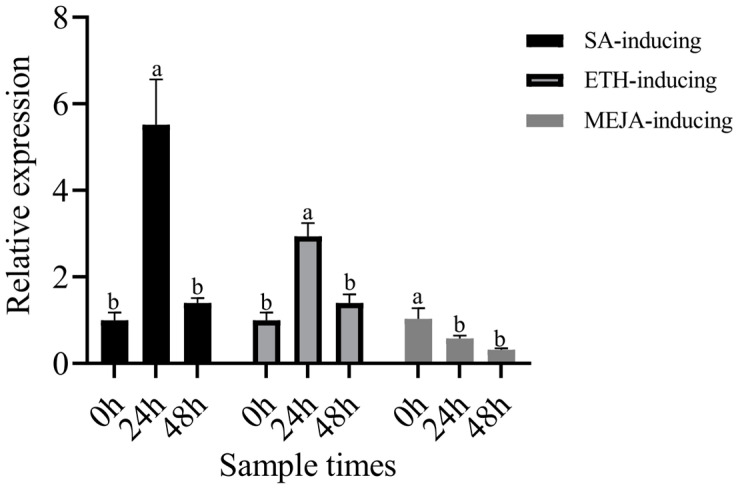
Relative expression analysis of the hub genes under SA, ETH, and MeJA induction. The error bars represent ±SD values, and different letters indicate significant differences between the two columns (*p* < 0.05).

ISR and SAR depend on the ET/JA- and SA-mediated signaling pathways, respectively. In order to investigate the induction of *RPS25* by these pathways, the expression of *RPS25* was performed by exogenous hormone inducing. The RT–qPCR results revealed that the expression of the hub gene *TRINITY_DN48668_c0_g2* (*RPS25*) was upregulated by SA and ETH, and its expression peaked at 24 h. Meanwhile, the expression of *RPS25* was downregulated by MeJA (Figure 12). 

## 4. Discussion

Conventional transcriptome analysis and WGCNA analysis have been applied to understand the host responses induced by *Trichoderma* inducing [26,27]. In this study, we used transcriptome analysis and the WGCNA analysis approach to identify resistance pathways and hub genes of *A. mongholicus* against *F. solani* elicited by *T. harzianum*. According to the pathways information, the determination of H_2_O_2_ content and formononetin and biochanin A inhibiting *F. solani* in vitro showed defense-related gene *PR1*, the production of H_2_O_2_, formononetin biosynthesis, biochanin A biosynthesis, *CHIB*, and *HSP90* were the elements that ultimately respond to *T. harzianum* induction. WGCNA analysis identified one core hub gene, *RPS25*, which was subsequently proven to respond to hormone-induced expression.

### 4.1. Induction of PR1 by T. harzianum under F. solani Stress

Calcium (Ca^2+^) is a common second messenger and changes in the Ca^2+^ concentration are among the earliest events following the induction of the establishment of plant–microbe symbioses, pathogen defense responses, and stress adaptation [55,56]. Transient increases in intracellular Ca^2+^ levels and defense responses are activated by metabolite mixtures secreted by the biocontrol fungus *T. atroviride* [57]. The hydrophobin HYTLO1 secreted by *T. longibrachiatum* also plays an important role in the early stages of the plant–fungus interaction and influences the Ca^2+^ level for 30 min, which then activates defense-related genes [58]. In this study, the defense-related gene *PR1*, which is regulated by the Ca^2+^ pathway, was upregulated in the three comparison groups. PR proteins offer protection against pathogenic infections by accumulating locally in the affected and surrounding tissues [59]. PR1 expression has been proven to be induced in the roots of potato plantlets by *T. harzianum* Rifai MUCL 29707 after 168 h and by Arabidopsis root colonization by *T. harzianum* after 48 h [60,61]. These findings indicate that the induction of *PR1* by *T. harzianum* may be involved in *A. mongholicus* resistance to *F. solani*. *CaMs*/*CMLs* act as crucial Ca^2+^-binding sensors that were also induced by *T. harzianum* in our study. The expression of *NbPR1* was significantly upregulated in 35S::*AhCML69* tobacco leaves infected with *R. solanacearum* [62]. We hypothesized that *CaMs/CMLs* may regulate the expression of *PR1* genes.

In addition, the expression of *Rd19*, which is involved in the plant–pathogen interaction pathway, and *NPR1*, which is involved in the plant hormone signal transduction pathway, were also upregulated by *T. harzianum*. In Arabidopsis, overexpression of *Rd19* displayed resistance to multiple pathogens. Rd19 protein activity may be activated through SA accumulation and, then, induced *PR* gene expression [63]. The *NPR1* gene is central to the activation of SA-dependent defense genes and is required for the control of Pythium ultimum infection by *T. harzianum* strain T22 in Arabidopsis seedlings [17,64]. The nuclear localization of NPR1 was found to be required for the activation of *PR* gene expression [65]. These results indicate that the *Rd19* and SA signaling pathway may regulate the expression of the *PR1* gene. 

Interestingly, SA can increase the endogenous Ca^2+^ level [66], and both of the regulatory pathways involved can induce the expression of *PR* genes. This phenomenon suggests that *PR* genes by *T. harzianum inducing* may play a key role in *A. mongholicus* resistance to *F. solani*. In addition, the expression of *PR* genes is a good molecular marker for the activation of SAR. These findings indicate that SAR can be activated by *T. harzianum* induction.

### 4.2. H_2_O_2_ Induction by T. harzianum under F. solani Stress

In our study, *CDPK* and *Rboh* were upregulated only in T + F_24 h compared with F_24 h. CDPKs are one of the Ca^2+^ sensors [66] and can activate the N-terminal EF-hands of Rboh [67]. Rbohs are plant NADPH oxidases that are responsible for ROS generation [68]. H_2_O_2_ assays revealed that *F. solani* infection decreased the accumulation of H_2_O_2_ at 24 h, but *T. harzianum* slowed the decrease in H_2_O_2_. ROS generation can promote programmed cell death (PCD) to restrict invading pathogens to infection sites [69]. With the infection of *F. solani*, the accumulation of H_2_O_2_ increased at 48 h, but the presence of *T. harzianum* decreased the increase of H_2_O_2_. Excessive production of ROS can cause damage to DNA, proteins, and lipids [70]. These findings indicate that the Ca^2+^ pathway by *T. Harzianum* inducing regulates ROS generation, facilitating resistance to *F. solani* infection. 

### 4.3. Formononetin and Biochanin A Biosynthesis Pathways Induction by T. harzianum under F. solani Stress

Phenylpropanoid, flavonoid, and isoflavonoid biosynthesis pathways were enriched in this study. Previous studies have shown that phenylpropanoid compounds are precursors to flavonoids and isoflavonoids [71]. Formononetin and biochanin A are plant isoflavones. In our study, formononetin and biochanin A can be regulated by three pathways together in T + F_24 h. These results indicated that the biosynthesis of formononetin and biochanin A can be activated by *T. harzianum*. Formononetin, when present at high levels, acts as an active fungicidal compound to protect plants against fungal diseases [72]. Biochanin A also showed high antifungal and antibacterial activity [73,74]. In the present study, formononetin and biochanin A have indeed been shown to have the potential to inhibit *F. solani* growth in vitro. And the inhibitory capacity of biochanin A was greater. When the concentration of biochanin A was 1 mg/mL, the growth of *F. solani* was significantly inhibited. However, whether the inhibitory concentration can be attained in *A. mongholicus* under T + F treatment is to be further investigated. 

### 4.4. CHIB Induction by T. harzianum under F. solani Stress

In our study, the expressions of *NDK*, *ACS6*, *ERF1*, and *CHIB* in the MAPK signaling pathway and the plant hormone signal transduction pathway were upregulated by *T. harzianum*. The expression of the gene *OsNDPK1* (*NDK*) in rice was also induced by bacterial pathogen infection and SA [75]. NDK can enhance the phosphorylation of activated MPK3 [76]. MPK3 is also capable of phosphorylating ACS6 and ACS2. ACS is the rate-limiting enzyme that catalyzes ET biosynthesis. *ACS6* and *ACS2* can be mutated to reduce B. cinerea-induced ET production [77]. *ERF* genes, which activate the expression of downstream ET-responsive genes, were upregulated by *T. harzianum* T22 [78,79]. While *CHIB* is an ERF target gene, the expression of *CHIB* was increased in *AtERF14*-overexpressing plants [80]. When plant (host) cells are subjected to pathogen stress, plant chitinases are highly expressed and thereby play a crucial role in combating fungal pathogens [59]. These results indicate that the induction of the defense-response gene *CHIB* by *T. harzianum* plays a key role in the resistance of *A. mongholicus* to *F. solani*. As *CHIB* was expressed in T + F_0 h vs. F_0 h and T + F_24 h vs. F_24 h, it is active mainly early in the process. The expression of *CHIB* depends on the ET signaling pathway, which showed that ISR can be activated by *T. harzianum* induction.

### 4.5. MYC2 and JAZ Induction by T. harzianum under F. solani Stress

MYC2 and JAZ in the JA signaling pathway were also induced by *T. harzianum* in our study. MYC serves as the fundamental regulator of the JA signaling branch, which is involved in plant development and multiple stresses [81]. The activation of JA signaling mediated by MYC2 is precisely regulated by its repressor JAZ and a subunit of the mediator complex (MED25) protein [82]. JAZ proteins are negative regulators of the JA signaling pathway [83]. In T + F_48 h vs. F_48 h, the expression of *MYC2* was upregulated; the expression of *JAZ* was the opposite. We hypothesized that *MYC2* induced by *T. harzianum* and activation of the JA signaling pathway were mainly effective in the later stage under *F. solani* stress. *MYC2* could be induced by *T. asperellum*-secreted protein *Epl1-Tas* [84] and was required for the ISR of the beneficial rhizobacterial strain *Pseudomonas fluorescens* WCS417r in *Arabidopsis thaliana* [85]. These manifestations suggested that the ISR dependent on the JA pathway can be activated by *T. harzianum.*

### 4.6. Candidate Hub Gene Mining under T. harzianum Treatment

A total of six genes, namely *TRINITY_DN48668_c0_g2* (*RPS25*), *TRINITY_DN43430_c0_g1* (*NDK*), *TRINITY_DN11766_c0_g1* (*Hsp90*), *TRINITY_DN8962_c0_g1* (*CYCD3*), *TRINITY_DN12696_c0_g1* (*LRR-RLK*), and *TRINITY_DN105680_c0_g1* (*CYPs*), were identified hub genes. Among the expression of *RPS25*, *NDK* and *Hsp90* were higher in the T + F treatment than in the F treatment. These three genes, by *T. harzianum* inducing, may play a major role in *A. mongholicus* against *F. solani.*

*TRINITY_DN48668_c0_g2* (*RPS25*), encoding the 40S ribosomal protein S25-2-like, was identified. Ribosomes and ribosome-associated proteins are required for protein synthesis [86,87]. The 40S small subunit, which is formed by small ribosomal proteins (RPSs) and 18S rRNA, is part of the eukaryotic ribosome [87,88]. A previous study revealed that the RPS6 mRNA expression and protein levels in Nicotiana benthamiana increased during infection with the turnip mosaic virus (TuMV), tobacco etch virus (TEV), and tobacco mosaic virus (TMV) [89,90]. The *RPS21* gene in tomato was upregulated by *T. harzianum* strain T22 treatment [78]. The increase in protein expression revealed that the expression of *RPS25* in Vanilla planifolia Jacks is an early plant response to *Fusarium* infection [91]. In this study, *RPS25*, which acts as a hub gene, was identified via WGCNA, and its expression was greater in the T + F treatment than in the F treatment overall. On the basis of the above, we assumed that *T. harzianum* can activate ISR and SAR. We also found that *RPS25* could be induced upregulation by the exogenous hormones SA and ETH. These results indicated that the induction of *RPS25* by *T. harzianum* may play a key role in the resistance of *A. mongholicus* to *F. solani*. 

Overall, the expression of *NDK* was greater in the T + F treatment than in the F treatment. *NDK* plays an important role in regulating the *CHIB* gene. This finding indicates that *CHIB* can be reliably used as an important resistance-related gene. 

Heat-shock proteins (Hsps) were triggered by virtually all stresses. *Hsp90*-located cytoplasm is accountable for pathogen resistance through interacting with the resistance protein [92]. It also can be induced by the *T. harzianum* strain M10 [22] and *T. harzianum* T39 [93]. In our study, the *Hsp90*-enriched plan–pathogen interaction pathway was also induced by *T. harzianum* and belonged to highly connected hub genes. We conjectured that *Hsp90* induced by *T. harzianum* positively regulated *A. mongholicus* resistance to *F. solani.*

## 5. Conclusions

*T. harzianum* has the potential to induce plant resistance against pathogens [94]. However, the molecular mechanism by which *T. harzianum* induces resistance to *A. mongholicus* is unclear. In this study, a total of 6361 DEGs in *A. mongholicus* were induced by *T. harzianum*. Analysis shows that resistance of *A. mongholicus* against *F. solani* depends mainly on pathways that regulate the defense-related gene *PR1*, the production of H_2_O_2_, formononetin biosynthesis, biochanin A biosynthesis and *CHIB*, and the gene *HSP90*. WGCNA revealed one core hub gene, *RPS25*, responding primarily to the induction of the exogenous hormones SA and ETH. This study contributes to a better understanding of the molecular mechanisms underlying the resistance induced by *T. harzianum* against *F. solani* in *A. mongholicus* roots. The genes identified from this experiment will be useful for selecting candidates for resistance to *F. solani* in *A. mongholicus*. The selected genes can be functionally validated by molecular biological methods.

## Data Availability

The original contributions presented in the study are included in the article/Supplementary Material.

## Supplementary Materials

The following supporting information can be downloaded at https://www.mdpi.com/article/10.3390/genes15091180/s1: Figure S1: Functional annotation of 6361 DEGs. (A) GO annotations analysis. (B) KEGG annotations analysis; Figure S2: Validation results of RNA-Seq by RT-qRCR for four genes; Table S1: Primers used for RT-qPCR; Table S2: A statistical summary for all RNA-Seq samples; Table S3: Statistical information of differentially expressed genes; Table S4: GO annotation analysis of *Astragalus mongholicus* 4224 DEGs for *Trichoderma harzianum* T9131 inducing; Table S5: Expression information of DEGs in key KEGG pathways; Table S6: Information of 58 connected hub genes.
