# Supplementary material for: Integration of Transcriptomics and WGCNA to Characterize Trichoderma harzianum-Induced Systemic Resistance in Astragalus mongholicus for Defense against Fusarium solani
Source: Genes (Basel). 2024 Sep 8;15(9):1180. doi: 10.3390/genes15091180 (PMC11431081; doi:10.3390/genes15091180)
Supplement: Supplementary file 1 [file genes-15-01180-s001.zip › Supplementary figure.pdf]

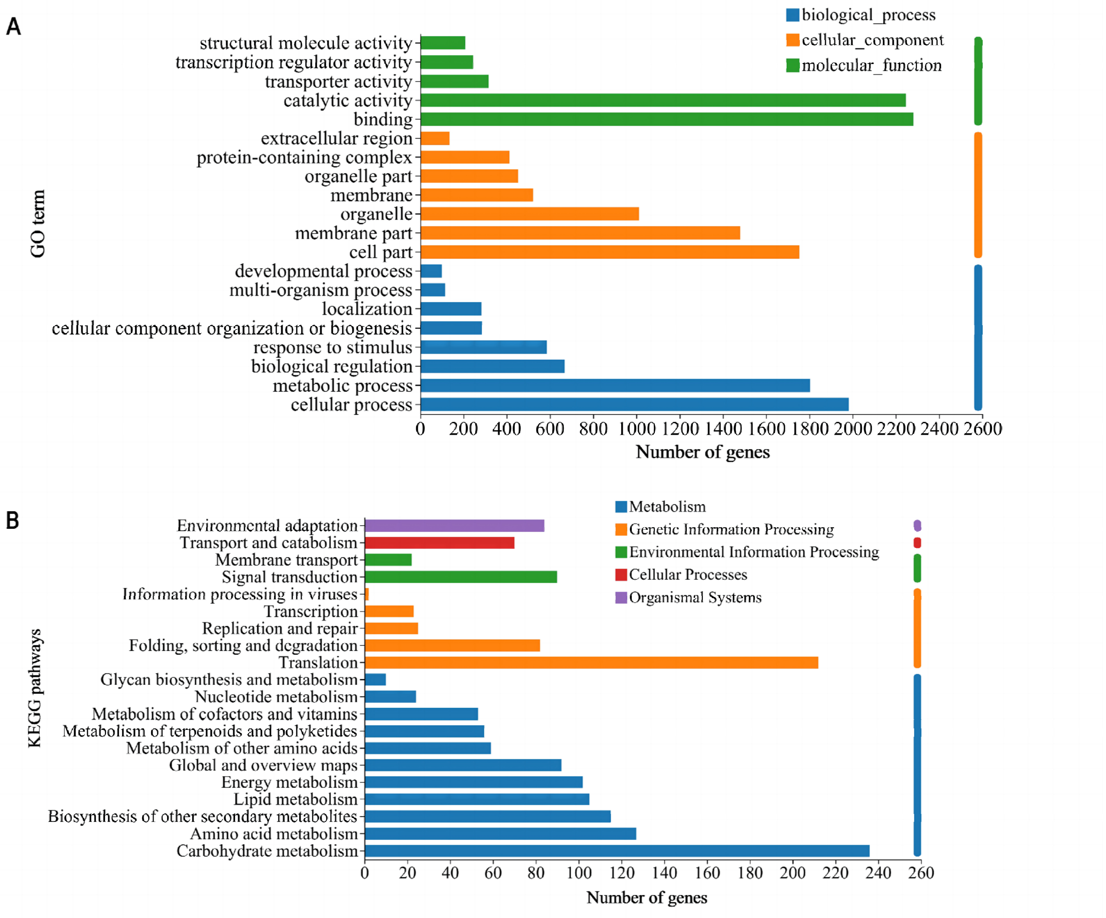

Figure S1. Functional annotation of 6361 DEGs. (**A**) GO annotations analysis. (**B**) KEGG annotations analysis.

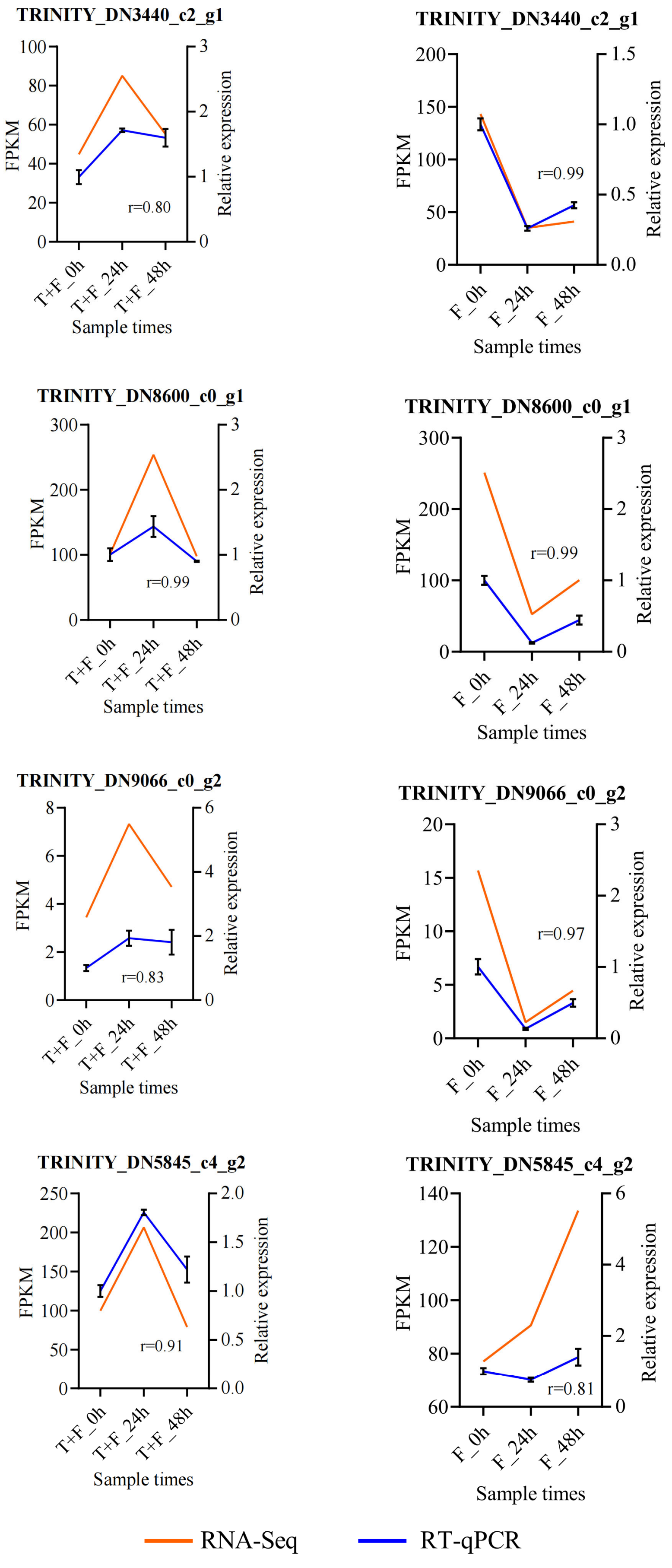
TRINITY_DN3440_c2_g1
FPKM
Relative expression
r=0.80
T+F_0h
T+F_24h
T+F_48h
Sample times
TRINITY_DN3440_c2_g1
FPKM
Relative expression
r=0.99
F_0h
F_24h
F_48h
Sample times
TRINITY_DN8600_c0_g1
FPKM
Relative expression
r=0.99
T+F_0h
T+F_24h
T+F_48h
Sample times
TRINITY_DN8600_c0_g1
FPKM
Relative expression
r=0.99
F_0h
F_24h
F_48h
Sample times
TRINITY_DN9066_c0_g2
FPKM
Relative expression
r=0.83
T+F_0h
T+F_24h
T+F_48h
Sample times
TRINITY_DN9066_c0_g2
FPKM
Relative expression
r=0.97
F_0h
F_24h
F_48h
Sample times
TRINITY_DN5845_c4_g2
FPKM
Relative expression
r=0.91
T+F_0h
T+F_24h
T+F_48h
Sample times
TRINITY_DN5845_c4_g2
FPKM
Relative expression
r=0.81
F_0h
F_24h
F_48h
Sample times
RNA-Seq
RT-qPCR

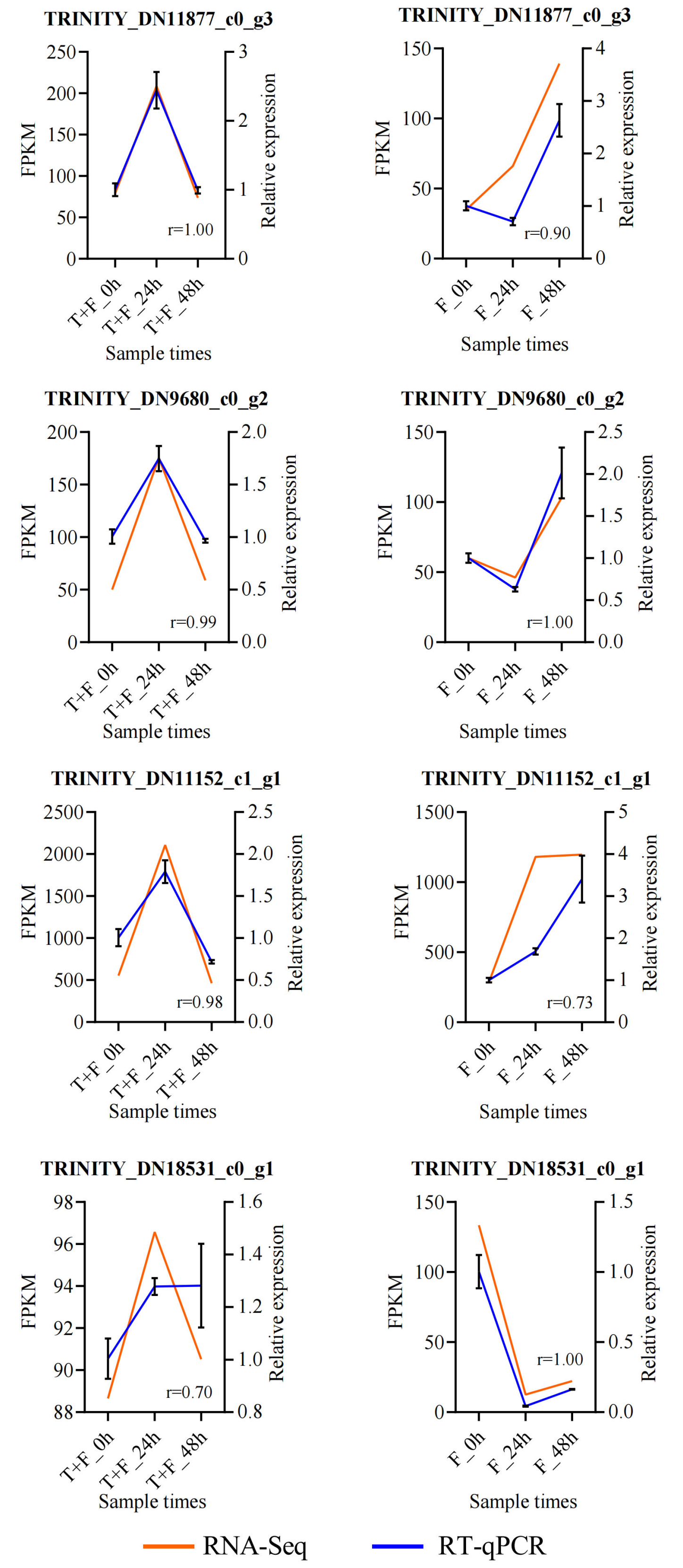

Figure S2. Validation results of RNA-Seq by RT-qRCR for eight genes. The left vertical axis indicates the FPKM value obtained via RNA-Seq, and the right vertical axis indicates the relative expression level determined via RT-qPCR. r values are the correlation coefficients between RT-qRCR and RNA-Seq.
